# Social Science Pedagogy as a Way of Integrating Sustainable Education and Global Citizenship into the Initial Training of Pre-Primary Teachers

**DOI:** 10.3390/ejihpe11030072

**Published:** 2021-08-26

**Authors:** Rocío Diez, Andrea Dominguez, Santiago Ponsoda, Bárbara Ortuño

**Affiliations:** 1 Department of General Didactics and Specific Didactics, Faculty of Education, University of Alicante, 03690 Alicante, Spain; santiago.ponsoda@ua.es (S.P.); barbara.ortuno@ua.es (B.O.); 2 Faculty of Education, University of Alicante, 03690 Alicante, Spain; adg36@alu.ua.es

**Keywords:** social sciences, critical thinking, higher education, teacher training, teacher training for pre-primary education, gender perspective, women’s history, family trees, feminism, global citizenship

## Abstract

The main objective of this research was to demonstrate the integration of international and national strategies in education for sustainable development and global citizenship into initial teacher training. The researchers analyzed the outcomes of a feminist teaching strategy based on family trees, focusing on the usefulness of social science pedagogy in developing critical thinking among pupils. They also attempted to enhance teachers’ digital literacy and make progress in reflecting on its functioning and use. The research used mixed methods, and the research instrument was a questionnaire using Likert-type scales validated by specialists from several universities. It was deployed in the module Didáctica del Conocimiento del Medio Social y Medioambiental (Pedagogy of Knowledge of the Social and Cultural Environment) of the Bachelor’s Degree in Pre-Primary Education at the University of Alicante. Using historical research with a gender perspective, the trainee teachers investigated their family trees, focusing on the women in their families. They also carried out a speculative exercise to aid reflection on their contributions as teachers in support of equal education. The results obtained showed that this was a novel and useful educational activity, which inspired participants to work for a fair and democratic global citizenship based on coeducation.

## 1. Introduction

Social science pedagogy must educate students in fostering critical thinking based on social justice and democracy in order to build the foundations for democratic citizenship [1]. Students’ critical understanding of an increasingly diverse society [2] needs to be nurtured—a critical understanding based on social justice, promoting active, responsible participation in constructing social alternatives [3,4,5].

Training in critical thinking is a useful strategy for responding to the current crisis in education. Traditional teaching methods, based on one-way transmission of knowledge and rote learning, generate mechanical responses, decontextualized from pupils’ lived realities. By contrast, a critical social science pedagogy will allow a connection with that complex reality. Many pressing social issues today (such as the resurgence of extreme nationalism, xenophobic and homophobic policies, forced migrations, the deepening of social, gender-based, and economic inequalities, and the climate emergency) are reaching young people through media and social networks, with all the shortcomings of these channels, and often without young people being able to select or interpret this information, much less connect it to what they learn in school, as [6] indicates. For these and other reasons, [7] says, education must contribute to strengthening society based on coexistence, solidarity, respect, and mutual aid. Social science methods and procedures strengthen and exercise the faculties of critical thinking and interpretative reasoning. They will, therefore, stimulate plurality and freedom of thought based on personal criteria and enhance students’ interpretative and analytical capacities by promoting active, agile, and diligent minds.

Social science pedagogy thus aligns itself with the international framework that defines the aims and objectives of education for our societies based on critical, responsible, and sustainable citizenship education, as set out in the 2030 Agenda for Sustainable Development [8], implemented through its 17 Sustainable Development Goals (SDGs).

The Education 2030 Framework for Action (FFA) also examines learning outcomes in relation to preparation for the labor market and preparation for life as an active citizen, as well as the traditional goal of equal access to education, set out in earlier programs such as Education For All (EFA) [8]. The target is that by 2030, all learners will acquire the knowledge and skills needed to promote sustainable development. The data show that it is precisely the countries with higher rates of school enrollment, better educational outcomes, and higher attainment that tend to have less sustainable lifestyles [9]. Fostering critical thinking about particular social customs is, therefore, essential to achieving sustainable development. 

The importance of education cuts across the 2030 Agenda. SDG4—the education goal—consists of 10 targets, but several other SDGs also include targets related to education, because of the high levels of interaction between SDGs [10]. This is why the 2030 Agenda envisages the goals being achieved simultaneously—progress toward SDG4′s targets is conditional on progress toward health (SDG3), gender equality (SDG5), ending poverty (SDG10), promoting a culture of peace (SDG16), and the other SDGs. Target 4.7 of SDG4 calls on countries to “ensure that all learners acquire the knowledge and skills needed to promote sustainable development, including, among others, through education for sustainable development and sustainable lifestyles, human rights, gender equality, promotion of a culture of peace and non-violence, global citizenship and appreciation of cultural diversity and of culture’s contribution to sustainable development”.

When the Education Framework for Action [8] evaluated the reality of education systems around the world in relation to the SDGs, it concluded that not all types of education will be able to achieve the SDG4 targets and that most education systems need to adapt to the skills required by current digital technologies. It made a particular call for education systems to equip learners with values for global citizenship. All these aspects must be taken into consideration at every level of education. Initial teacher training in universities stands out as of fundamental importance, however, since it involves the citizens whose job it will be to educate the children and young people in every society, and whose responsibility it will be to educate for fairer, more inclusive, and more sustainable societies. It is, therefore, desirable to introduce cross-cutting competencies into university curricula to meet these objectives [11]. We agree that higher education is the setting where education for fair and sustainable social development can best be delivered [12]. Critical and responsible participation of students in the changes that will drive equality and justice must be encouraged [13].

Global citizenship education, a key issue in UNESCO’s Education Framework for Action, was already being considered in the Preamble to UNESCO’s Constitution (1945), to “build peace in the minds of men and women” [14]; in the Universal Declaration of Human Rights (1948) [15]; in the Recommendation concerning Education for International Understanding, Co-operation and Peace and Education relating to Human Rights and Fundamental Freedoms (1974) [16]; in the World Programme for Human Rights Education (2005 to the present date) [17]; and of course, in the Education 2030 Agenda, as already discussed, primarily in target 4.7 of the Sustainable Development Goals.

Spanish legislation on education also urges us to foster critical thinking, as shown in Organic Law 3/2020, of 29 December, which amends Organic Law 2/2006, of 3 May, on Education [18]. This law reminds the reader that students should have a deep knowledge of the history of democracy in Spain, from its origins up to the present day, from a gender perspective reflecting the feminist struggle and women’s struggle to achieve citizenship. It states that “the study and analysis of our democratic memory will allow civic values to be embedded and will contribute to the education of freer, more tolerant citizens with critical minds”.

If we consider the different stages of education, with the new law on education entering into force in Spain, the importance of implementing the Education 2030 Agenda and SDG4 in pre-primary education (0–6 years) is evident. Similarly, efforts are being made to encourage social relationships on a footing of gender equality, the progressive acquisition of basic guidelines for coexistence, and exercises in empathy and peaceful resolution of conflicts, avoiding violence. Education in values, education for responsible and sustainable consumption, and health education and promotion are all included in both the first and second cycles (0–3 years and 3–6 years, respectively) of pre-primary education.

In primary education (6–12 years), education in civic and ethical values will be included in the curriculum in one of the academic years forming the third cycle (10–11 years and 11–12 years). It is as part of this subject that pupils will be taught content related to the Spanish Constitution, awareness of and respect for human rights and children’s rights, education for sustainable development and global citizenship, gender equality, the value of respect for diversity, and the social value of taxes. A critical mind and a culture of peace and non-violence will be promoted. For compulsory secondary education, which covers ages 12–16, Article 14 of the law mentioned above sets out the following objectives: “Developing basic skills in using sources of information to acquire new knowledge with a critical mind. Developing basic technological competencies and making progress in ethical reflection on their functioning and use”.

With regard to the curriculum, we should bear in mind that the “España 2050” report [19] published by the Spanish government points out the need to continue making progress in learning through skills. The objective is to break out of teaching organized around too large a number of subjects, which in turn are overloaded with content and too rigidly confined by textbooks, and moreover compete with each other for pupils’ attention, despite the efforts made in recent laws on education in Spain. We note that, in the absence of the changes required, an educational curriculum like the Spanish one will generate unidirectional, non-experiential, academic knowledge, based on non-interdisciplinary learning that hampers the development of basic cross-cutting skills such as teamwork, the ability to construct an argument, assertiveness, and critical thinking, as pointed out in the PISA report [20]. This is why “España 2050” outlines changes to the way the educational curriculum is conceived, designed, and implemented. It proposes to exercise the capacities required to develop critical thinking, creativity, and futures thinking, as well as how to express these orally and in writing, as indicated in UNESCO’s Futures Literacy report [21].

“España 2050” also shows that Spanish society is below the European average in core subjects of vital importance in people’s personal and professional development, such as knowledge of foreign languages, digital skills, financial literacy, and mastery of cross-cutting or soft skills such as critical thinking, creativity, and curiosity. This is why it is essential to bring universities closer to the country’s economic and social fabric, updating the curricula of undergraduate and postgraduate degrees to develop the competencies that students will require in their professional careers. To achieve this, the starting point will be a more generalist perspective encouraging the acquisition of cross-cutting competencies such as written comprehension, verbal communication, and critical thinking, which help create more diverse profiles and widen people’s employability.

We believe it is possible to educate with the aim of generating critical thinking that will endow students with the capacity to reason, as described in the doctoral thesis, “La educación para la ciudadanía democrática y la Didáctica de las Ciencias Sociales. Estudio de un caso de investigacion-acción en la formación inicial de maestros de Educación Primaria” (Global Citizenship Education and Social Science Pedagogy: A Case Study of Action Research in the Initial Training of Primary Education Teachers) [22]. Social science pedagogy is the perfect setting for this, since critical thinking is part of the set of values and processes transmitted in the subjects forming this domain, from education for citizenship and human rights to social science pedagogy, passing through the pedagogy of knowledge of the social and cultural environment. Teaching strategy must therefore be directed at transforming traditional, more transmissive and unidirectional practices, so that students are provided with the tools, the information, and the opportunities they need to develop and train their critical thinking abilities. These requirements are a perfect match for the experiences provided by the social sciences, such as researching history and geography, and understanding behaviors, facts, relevant processes, and actors participating in events, whether individuals or groups, well-known or anonymous. Within social science pedagogy, the feminist perspective and coeducation have already demonstrated their effectiveness in preventing various kinds of violence or inequality by emphasizing respect for individuals, as educating for gender equality is educating for peace and citizenship [23,24,25,26].

The information and communication society (ICS) has likewise undoubtedly revolutionized education, creating new models and methods of teaching and learning such as e-learning [27]. This has increased teachers’ interest in employing information and communication technologies (ICTs) during classes [28]. ICTs are considered to be a set of instruments that allow pupils to acquire attitudes, skills, and processes, thereby generating meaningful learning [29]. ICTs and their innovative contributions constitute a revolution at all stages of education [30], but it is in higher education that teachers—including those still in training—are expected to have sufficient digital competence to adapt to the use of the new technologies in their areas of expertise [31]. 

For Santisteban [32], Dewey and Freire say that education is not a natural process but a form of social control, according to which social studies are defined when the kind of society we want to achieve is defined. In the works of Freire—essential reference works in critical pedagogy—education can be understood in two different ways, either as an instrument to facilitate people’s integration into the logic of the social system and bring about their conformity to it, or as a process for the “practice of freedom”. We endeavor to put ourselves on the side of the “practice of freedom”, and we are conscious that educational practice is fundamental: “We deal with people [...]. We participate in their development. We may help them or set them back in their search”. [33] In Freire’s terms, “Incompetence, poor preparation, and irresponsibility in our practice may contribute to their failure. But with responsibility, scientific preparation, and a taste for teaching, with seriousness and a testimony to the struggle against injustice, we can also contribute to the gradual transformation of learners into strong presences in the world”. [33] The current educational context and the immediate future demand this of us.

Teaching strategies to achieve this learning should integrate certain characteristics. Some studies seem to indicate that the didactic strategies traditionally employed in the social sciences are more in line with Education for Sustainable Development [34]. It is also known that, when the curricula of their initial training include subjects or content on critical citizenship, teachers feel more confident to address these issues throughout their professional lives [35]. Classroom climates that are open for discussion and allow respectful questioning of ideas are effective in promoting positive civic development [36], and knowledge- and social justice-based methods are suggested [37].

In order to build critical and democratic citizenship through the teaching of Social Sciences, it is important to use interactive didactic strategies that use testimonial resources, with an emotional base related to the students’ experiences [38], as well as considering the research [39], which defends the role of emotional competencies in supporting a global citizenship or development education perspective in student teachers’ practice.

Other didactic experiences with a gender perspective in initial teacher training show positive results, and recognize older women as the main transmitters of knowledge, especially those related to health and care [40]. The need to re-signify the method of genealogies from a feminine and feminist point of view is pointed out [41]. The re-establishment of genealogical links is a political strategy, and also an educational one, which makes it possible to recover women’s legacies and to emphasize the significance of what has happened in each historical moment, thus revisiting feminist thought and political action since its emergence [41]. The use of genealogy is a creative proposal, tremendously fruitful in feminist studies, and a possible way to answer who we are and how we came to be what we are [42]. The study of these genealogies “shows the links in the chain established between successive generations, reflects learning, traditions, strategies and transformations, and highlights the need to recover unknown remnants of an individual and collective memory” [43]. 

For all the reasons outlined above, a research study was designed in the domain of social science pedagogy, within the specific module 172016 Pedagogy of Knowledge of the Social and Cultural Environment of the Bachelor’s Degree in Pre-Primary Education, in the Faculty of Education at the University of Alicante. Considering the contributions of Action Research, an attempt was made to evaluate the usefulness of feminist genealogies in initial teacher training, as a strategy to promote critical thinking and to promote the use of feminist genealogies in teacher training. Its aims were (a) to incorporate international strategies in education for sustainable development and global citizenship into initial teacher training, (b) to use feminist didactic strategies to enhance the traditional usefulness of social science pedagogy in developing students’ critical thinking, and (c) to improve teachers’ digital skills and make progress in critical reflection on their functioning and use.

Our aim is for teachers to encourage research into the meaning and scope of real equality between women and men, with the ultimate goal of achieving social justice. To this end, a practical activity has been designed based on the creation of a family tree, but from a gender and women’s history perspective, with the aim of paying special attention to the life stories of the women in the students’ families.

## 2. Materials and Methods

A mixed-methods approach was chosen for this research. This uses a combination of different research approaches or multiple methodological strategies to explore and answer questions on a specific topic. It can be conceptualized as a deliberate combining of qualitative and quantitative research approaches [44], in order to tackle a research problem more effectively [45].

### 2.1. Research Context and Participants

The research was undertaken in the Faculty of Education at the University of Alicante (Spain), among students taking the module Pedagogy of Knowledge of the Social and Cultural Environment of the Bachelor’s Degree in Pre-Primary Education. A total of 283 students took part in the research, 92.2% of whom (*n* = 261) were women and 7.8% (*n* = 22) were men. The age distribution of the participants was 88.7% under 25 years old; 5.7% between 26 and 30; 1.4% between 31 and 35; and 4.2% over 35. All subjects gave their informed consent for inclusion before they participated in the study.

### 2.2. Instrument

The instrument used in this research was designed specifically to analyze students’ opinions and ratings of a teaching strategy and was validated by experts from the universities of Burgos, Salamanca, and Murcia. In addition to questions about sociodemographic characteristics, 8 question items were created using a Likert scale from 1 to 5, where 1 means “strongly disagree”, 2 means “disagree”, 3 means “neither agree nor disagree”, 4 means “agree”, and 5 means “strongly agree”. The 8 questions addressed in this first part were: (1) The realization of life stories has revealed to me significant information that I did not know, (2) I consider this practice useful for my personal training, (3) I have done a similar practice before, (4) The learnings obtained in this practice inspire me for my future teaching practice, (5) I can do many things for a more coeducational education, (6) Thanks to this activity I have improved my consideration of the need to include activities with a gender perspective in my future professional practice, (7) I am willing to get involved in the fight for gender equality in my future professional practice, and (8) Improvements in coeducation will depend in part on my professional performance.

The questionnaire also included 3 open questions to allow students to give their opinions on the positive and negative aspects of the educational activity and on the learning they felt they had acquired through the teaching strategy.

The data from the Likert scale questions were analyzed using IBM SPSS v. 25 for Windows. Firstly, the internal consistency of the questionnaire was tested using Cronbach’s alpha, with a result of 0.685. For scales with fewer than 10 items, a Cronbach’s alpha value of 0.6 or above is acceptable [46], so the result obtained for the questionnaire used in the study suggests a good level of internal consistency (Table 1).

The open questions were analyzed by coding the responses. MAXQDA software was used for this qualitative analysis, and, based on the classification categories created, a code map was set up, which was then validated by experts in educational and qualitative research.

### 2.3. Procedure

The study investigated a teaching strategy involving family trees, which was closely linked to developing students’ critical thinking and digital teaching skills. Among the didactic objectives of the activity, the following stand out: (a) to analyze life stories from a gender perspective, (b) to detect gender inequalities that have conditioned life trajectories, (c) to learn about and reflect on the job expectations in terms of co-education and equality of future Early Childhood Education teachers.

The specific activity chosen was a practical one titled “The women in my tree: Putting down roots for the future”, which consisted of creating a free-format family tree covering at least three or four generations. This was an individual and compulsory activity for the students taking the course in the continuous evaluation modality. We then asked the participants to choose one of the women in their family and reconstruct her life course through an in-depth oral interview, focusing on the aspects or topics that the participants considered relevant to demonstrating sex or gender differences, for example, in access to education or in their working lives, in their experiences during the Spanish Civil War (1936–1939) or the Franco dictatorship (1939–1975), in their leisure activities, or in childcare. Lastly, we challenged the participants to write a brief speculative text in which they imagined their future, specifically the days immediately preceding their retirement, when they would remember their actions, behaviors, and attitudes in support of coeducation and a more egalitarian world. 

Once the activity was completed, the researchers sent the participating students the questionnaire via Google Forms and collected the information to be analyzed.

## 3. Results

### 3.1. Quantitative Results 

In Table 2, we see that for item 1, “Collecting life stories has revealed significant information I was not previously aware of”, 42% of students totally agreed that they had collected significant information, compared with only 2.1% who strongly disagreed. The average value for this item was 4.04 out of 5. 

The second item asked students about the usefulness of this kind of practice for their personal development (Table 3). More than half (55.5%) fully indicated that it was a useful educational activity, compared with 0.7% saying they did not consider this type of activity to be useful at all. The average value for this question was 4.38 out of 5.

Students were asked if they had carried out a similar kind of practice prior to this (Table 4). Only 9.5% of students participating said they had, compared with 39.9% who had not. The average value was 2.25 out of 5.

On the question asking students whether the learning they had achieved provided them with inspiration for their future work as teachers, 58.7% gave a strongly positive answer, while 0.7% did not consider this activity relevant to their future professional practice (Table 5).

Item 5 asked the future teachers whether they could work to support more coeducational education, and 68.9% of participants responded that they completely thought they could (Table 6).

The next question asked participants whether the activity had influenced their consideration of the need to include more activities with a gender perspective in their professional futures. A majority of 60.4% answered that it had had a positive impact on them, while 0.7% thought it had not had an impact on their consideration of the need to include such activities (Table 7). The average value was 4.47 out of 5.

When students were asked about their willingness to be involved in the struggle for equality and coeducation, 88.7% responded entirely favorably, while 0.4% showed fully disagreement with this statement (Table 8). The average value was 4.86 out of 5.

The final quantitative item was about whether students believed that improvements in coeducation would partly depend on their performance as teachers, and 66.1% fully thought that this was the case, while 1.1% did not share this view (Table 9). The average value was 4.58 out of 5.

### 3.2. Qualitative Results 

Among the qualitative findings, we highlight the fact that 35.9% mentioned “getting to know things I did not know before about the women in my family” and 28.6% mentioned “getting to know my family”. We are aware that the frenetic times we are living through and the new ways of having relationships with family members have been undergoing transformation for more than a decade. Increasing individualism, the way technology has monopolized social gatherings, and the decline in the perceived importance of older generations, among other things, have led to communication being in short supply or superficial in nature for many families in the Western world. We must also take into account that this teaching strategy was implemented against the backdrop of a global pandemic, which increased the isolation experienced by older people—we must not forget that the vast majority of students opted to interview their grandmothers—and, in many cases, these interviews were carried out by phone or video call after months of isolation. In our opinion, the exceptional emotional regime prevailing between interviewers and interviewees [47], on top of the exercise in empathy that research involving oral sources always entails, undoubtedly fueled students’ tendency to give positive evaluations of this practice. We present this reflection by one of the participating students by way of illustration:

“First of all, I’d like to thank everyone responsible for organizing the practices, given the connection it led me to experience. Because of the pandemic, my grandmother has not been able to leave the home all year, and phone calls, the poems she was writing, and what she observed through a window enabled her to get through the days. Calling her to reconstruct her life story was a pleasure not just for me but for her too, as she was able to reassert herself and recognize herself through memory as what she is, a brave, determined, and hardworking woman. I will always be grateful for this.”

Another student wrote in a similar vein:

“It was a real pleasure to carry out this activity. Recalling memories, discovering stories about the past, finding old photos, feeling lucky to have my great family, and making a mural that is already hanging in my place of work: these are some of the positives I got out of this activity at a personal level. So many thanks for proposing it.”

Table 10 shows that the positive aspect most commonly highlighted by students was getting to know things they did not know before about the women in their family (code 1.1), as we see illustrated in the following verbatim comments: (P048) “I discovered things my grandmother lived through that I didn’t know about before” and (P091) “Getting to know the female side of our family properly”.

In second place was getting to know the family (code 1.2), as illustrated in verbatim comments (P038) “It allows you to find out new things about your family” and (P062) “It has helped me get to know members of my family”. The importance of coeducation was also highlighted (code 1.3), seen in the verbatim comments (P015) “Reflecting on the importance of coeducation” and (P0168) “I realized how important coeducation is”.

Reflecting on the future (code 1.4) was also mentioned as a positive aspect and can be seen in verbatim comments (P0140) “I became aware of the things I would like to be and do throughout my life” and (P0147) “Imagining myself in the future in order to find out what I want to experience”.

Furthermore, the practice encourages gender equality (code 1.5), as students showed in verbatim comments (P0184) “We achieved an even deeper understanding of the importance of encouraging equality in education” and (P0196) “The importance of equality”.

Lastly, participants mentioned encouraging interaction with a loved one (code 1.6), as illustrated in verbatim comments (P0222) “I shared some special moments with my grandmother because of this activity” and (P0241) “I spent some pleasant time with my mother remembering moments from her childhood and her life in general”.

When asked about the negative aspects of the practice (Table 11), the most common response from students was that they did not encounter any difficulties (code 2.1), as seen in verbatim comments (P04) “No negative aspects” and (P031) “No negatives”. However, other responses highlighted difficulties with the interview (code 2.2), as illustrated in verbatim comments (P040) “I would like to have interviewed my grandmother, but it wasn’t possible because of the circumstances” and (P090) “Someone might find it tricky to carry out this interview because of family problems they do not want to discuss at work”.

Students also mentioned the difficulty of thinking about a future (code 2.3), seen in verbatim comments (P044) “Perhaps the last part of the work when you have to pretend you have already retired was a bit more difficult” and (P049) “I found it really hard to concentrate on what I wanted to do in the future”. Difficulties making the family tree (code 2.4) was another negative aspect mentioned, and this is seen in verbatim comments (P03) “When I tried to make the tree it was hard for me to find some of the photos” and (P0148) “Finding all the people on my family tree”. Similarly, the table shows some students had difficulty obtaining historical information (code 2.5), illustrated in verbatim comments (P0235) “Accessibility of documents” and (P0257) “Difficulty collecting some information”. Lastly, we see that students detected some difficulties related (in our opinion) to their conception of the practice (code 2.6), as illustrated in verbatim comments (P015) “The difficulty I encountered was the length of the life stories” and (P0154) “It is a very open practice, so lots of different perspectives are possible”.

Regarding the learning achieved (Table 12), students highlighted learning related to family experiences (code 3.1), as observed in the following verbatim comments: (P012) “Making discoveries about your own family” and (P020) “I learned the entire history of my family members”.

In second place they highlighted learning related to coeducation (code 3.2), illustrated by verbatim comments (P032) “Learning about coeducation which will help us to educate in equality” and (P044) “The importance of coeducation inside and outside the classroom”. Similarly, they pointed to learning about the role and evolution of women in recent history (code 3.3), seen in verbatim comments (P070) “Vision more focused on the change and evolution of the role of women in society” and (P0117) “We learned to value women and recognize their value”. They also pointed to historical learning (code 3.5), seen in verbatim comments (P010) “Understanding of the world and life 70 years ago” and (P0112) “Discovering historical facts”. In a similar vein, they mentioned learning related to gender equality (code 3.4), seen in verbatim comments (P014) “Understanding concepts related to equality” and (P0135) “Valuing equality more highly”.

Lastly, they highlighted learning about reflection and/or implementation of future perspectives (3.6), as illustrated by verbatim comments (P084) “I would highlight reflection on what my life will be like” and (P0153) “I learned to have great future vision”.

## 4. Discussion and Conclusions

As we pointed out at the start of our paper, social science pedagogy aligns itself with the international framework that defines the aims and objectives of education for our societies based on critical, responsible, and sustainable citizenship education, as set out in the 2030 Agenda for Sustainable Development [8], implemented through the 17 Sustainable Development Goals (SDGs). Spanish legislation on education also urges us to foster critical thinking, as set out in Organic Law 3/2020, of 29 December, which amends Organic Law 2/2006, of 3 May, on Education [18], and it recommends doing so from the foundations of society upward—in other words, from pre-primary education onward. The education of future teachers must play a key role in achieving this, if we want to ensure comprehensive early childhood education that includes education in values and education for responsible and sustainable consumption, emphasizing exploration of the environment where living creatures coexist and of the physical and social characteristics of that environment. Going one step further, this law adds a new paragraph encouraging teachers to “Promote, apply, and develop social norms that promote gender equality”. Gender equality is, of course, the foundation on which our research stands.

Piaget’s classic theories present pupils in pre-primary education as people who have great difficulty assimilating social, temporal, and spatial concepts because of the high level of abstraction of such concepts. The extension of these theories had the effect of discouraging research on this stage of education to a great extent and may have had an impact on the development of the cognitive and social capacities of children in this age range [48]. Nevertheless, the situation today is different, and this is demonstrated by the good results achieved in López Martínez’s education research practice based on social science pedagogy through the introduction of relevant social problems, specifically the presence of Syrian refugee children in a class of five-year-olds [39]. For López Martínez:

“*The future professional pre-primary teacher, placed on a globalized and uncertain stage, must understand that the academic knowledge they encounter in their different formal educational contexts will be conditioned by economic, social, political, and cultural circumstances. The latter will be marked by the hegemonic Eurocentric, androcentric, capitalist, and adult sociocultural model based on the rigid territorial framework of the nation-state, in spite of supranational projects*”.

[49]

Being conscious of these circumstances, of course, encourages critical thinking and thinking about social justice. In the case of the students who took part in “The women in my tree: Putting down roots for the future”, getting closer to their female genealogies and being conscious of the difficulties and violence—private and public—that their mothers, grandmothers, and great-grandmothers had to endure, for the simple reason that they were women, led to changes not just in their knowledge of the past and their family legacy, but in themselves, too. Understanding that their mothers, and more especially their grandmothers and great-grandmothers, are and were illiterate because they were deprived of an education due to the roles assigned to them by the established patriarchal system, made them reevaluate their choice to undertake teacher training. It also helped to empower them in respect of their studies, so little valued in general by society, and it led them to imagine a future of teaching influenced by the educational activities they carry out in support of coeducation and equality. 

Involvement in the design of an educational practice aimed at solving a relevant social problem or a live social issue (like the invisibility of women in history) and at introducing a gender perspective (here through a feminist methodology such as the family tree) has allowed us to provide trainee teachers with pedagogical tools that may, in the not-too-distant future, lead to practices designed using critical thinking, based on reflection, and aspiring to a comprehensive, democratic, egalitarian, fair, and engaged education from the foundations of society upward. The formative use of feminist genealogies through our students’ participatory research has strengthened their commitment to social justice and gender equality, while reinforcing their perceived ability to educate for equality and social justice [35,40]. These findings are in line with research from other Spanish Faculties of Education [11,22,32,35,40]. Undoubtedly, the participative didactic approach which focused on students’ experiences and emotions has contributed to these positive results [36,37,38,39]. 

The gratitude conveyed to us by the participating students via various channels is, of course, strongly connected with the investigation process itself experienced by the students as they carried out this practice. Discovering the “other” (female) actors in history, the ordinary people who contributed to the well-being of society through their daily tasks, despite all the obstacles put in their way by the patriarchy, produced emotions and pride in most of the participating students, in our opinion. It left visible traces on the teaching and learning process and on their empowerment as women and especially as future teachers in pre-primary education. In this connection, we would like to observe that the topic of masculinities in the Bachelor’s Degree in Pre-Primary Education and the influence of gender stereotypes on professional vocations linked to the social sciences are among the active research avenues of the Grupo de Investigación en Igualdad, Género y Educación (IGE) (Research Group in Equality, Gender, and Education) at the University of Alicante.

Through the results of our study, we are, in essence, affirming that the main aim of the teaching of social sciences must be to instill and develop in students a critical thinking based on social justice and democratic values. As we stated at the start of this article, the teaching of social sciences must itself be educated in social, critical, and creative thinking so that it can build the foundations of a democratic citizenship [1]. In critical thinking, we find a highly effective tool for responding to the crisis that (in our opinion) education is currently going through, as society in general is, too, as demonstrated by the dramatic rise of far-right parties and the increases in hate speech and violence against women, migrants, and members of the LGBTIQ+ community. We believe that if we manage to overcome the heavy bureaucratic burdens and the multitude of tasks imposed on university teachers, we will be able to continue researching, reflecting, and questioning students through our teaching practice—which is, after all, the ultimate goal of our profession—so that future teachers can become strong presences in the world and the true creators of a global citizenship that is democratic, critical, and fair in both social and environmental terms.

## Figures and Tables

**Table 1:** Internal consistency of the questionnaire used.

Reliability	Cronbach’s Alpha	Elements
Values	0.685	8

**Table 2:** Analysis of results for item 1: Collecting life stories has revealed significant information I was not previously aware of.

Values	Percentage	Average Value (between 1 and 5)
Value 1	2.1	4.04
Value 2	7.8	
Value 3	16.6	
Value 4	31.4	
Value 5	42	

Source: Prepared by the research team.

**Table 3:** Analysis of results for item 2: I consider this practice to be useful for my personal development.

Values	Percentage	Average Value (between 1 and 5)
Value 1	0.7	4.38
Value 2	1.8	
Value 3	11.7	
Value 4	30.4	
Value 5	55.5	

Source: Prepared by the research team.

**Table 4:** Analysis of results for item 3: I had already carried out a similar kind of practice prior to this.

Values	Percentage	Average Value (between 1 and 5)
Value 1	39.9	2.25
Value 2	25.1	
Value 3	14.8	
Value 4	10.6	
Value 5	9.5	

Source: Prepared by the research team.

**Table 5:** Analysis of results for item 4: The learning achieved during this practice provides me with inspiration for my future practice as a teacher.

Values	Percentage	Average Value (between 1 and 5)
Value 1	0.7	4.60
Value 2	1.8	
Value 3	8.5	
Value 4	30.4	
Value 5	58.7	

Source: Prepared by the research team.

**Table 6:** Analysis of results for item 5: I can do a lot of things to support more coeducational education.

Values	Percentage	Average Value (between 1 and 5)
Value 1	0.4	4.60
Value 2	0.7	
Value 3	6.4	
Value 4	23.7	
Value 5	68.9	

Source: Prepared by the research team.

**Table 7:** Analysis of results for item 6: The activity has increased my consideration of the need to include more activities with a gender perspective in my future professional practice.

Values	Percentage	Average Value (between 1 and 5)
Value 1	0.7	4.47
Value 2	1.1	
Value 3	9.5	
Value 4	28.3	
Value 5	60.4	

Source: Prepared by the research team.

**Table 8:** Analysis of results for item 7: I am willing to get involved in the struggle for equality and coeducation.

Values	Percentage	Average Value (between 1 and 5)
Value 1	0.4	4.86
Value 2	0.4	
Value 3	1.4	
Value 4	9.2	
Value 5	88.7	

Source: Prepared by the research team.

**Table 9:** Analysis of results for item 8: Improvements in coeducation will partly depend on my performance as a teacher.

Values	Percentage	Average Value (between 1 and 5)
Value 1	1.1	4.58
Value 2	0	
Value 3	5.3	
Value 4	27.6	
Value 5	66.1	

Source: Prepared by the research team.

**Table 10:** Positive aspects of the practice mentioned by students.

Codes	Mentions	% of Mentions
1.1 Getting to know things I did not know before about the women in my family	98	35.9
1.2 Getting to know my family	78	28.6
1.3 The importance of coeducation	31	11.3
1.4 Reflecting on the future	24	8.8
1.5 Encouraging gender equality	21	7.7
1.6 Encouraging interaction with a loved one	21	7.7

Source: Prepared by the research team.

**Table 11:** Negative aspects/difficulties of the practice mentioned by students.

Codes	Mentions	% of Mentions
2.1 No difficulties	91	27.8
2.2 Difficulties with the interview	69	21.1
2.3 Difficulties inventing the future	61	18.6
2.4 Difficulties making the family tree	45	13.7
2.5 Difficulties obtaining historical information	40	12.2
2.6 General difficulties with the practice	22	6.4

Source: Prepared by the research team.

**Table 12:** Learning achieved by students.

Codes	Mentions	% of Mentions
3.1 Learning related to family experiences	106	31.1
3.2 Learning related to coeducation	77	22.6
3.3 Learning related to the role and situation of women	50	14.7
3.4 Learning related to gender equality	48	14.1
3.5 Historical learning	36	10.5
3.6 Learning about future perspectives	24	7

Source: Prepared by the research team.

## Data Availability

The datasets generated and analyzed in the current study are available from the corresponding author upon reasonable request.
